# Recognizing stressed chicken signs: A comparison using the Happy Chicken Tool and the Stressed Chicken Scale

**DOI:** 10.1016/j.psj.2025.106141

**Published:** 2025-11-19

**Authors:** Larissa Schlegel-Pape, Robert Opitz, Marko Henning, Cristina Ortiz Cruz, Anne S. Kleine, Sabine G. Gebhardt-Henrich, Hans Mielke, Carola Fischer-Tenhagen

**Affiliations:** a Freie Universität Berlin, Faculty of Veterinary Medicine, Farm Animal Clinic – Division for Poultry, Königsweg 63, 14163 Berlin, Germany; b German Federal Institute for Risk Assessment, Unit Epidemiology, Statistics, and Mathematical Modelling, Department Exposure, Berlin, Germany; c Johann Heinrich von Thünen Institute, Federal Research Institute for Rural Areas, Forestry and Fisheries, Centre for Information Management, Bundesallee 44, 38116 Braunschweig, Germany; d Max Rubner-Institut, Federal Research Institute for Nutrition and Food, Zentralabteilung, Haid-und-Neu-Str. 9, 76131 Karlsruhe, Germany; e TAP Calanda AG, Chur, Switzerland; f Center for Proper Housing: Poultry and Rabbits (ZTHZ), Animal Welfare Division, Veterinary Public Health Institute, University of Bern, Zollikofen, Switzerland; g German Federal Institute for Risk Assessment, German Centre for the Protection of Laboratory Animals (Bf3R), Berlin, Germany

**Keywords:** noninvasive stress assessment, computer vision, automated stress detection, machine learning, transfer learning

## Abstract

This study investigates the use of deep learning, specifically convolutional neural networks (CNNs), and transfer learning for detecting signs of discomfort in chickens through image analysis.

We present a comprehensive framework that includes data preparation, model training, and evaluation using transfer learning with pre-trained CNN models such as EfficientNet and MobileNet. The methodology includes image extraction from video footage, followed by preprocessing, and augmentation to improve dataset diversity and robustness.

Model performance was evaluated using cross-validation on the original dataset and validation on two separate datasets, with metrics such as accuracy, sensitivity, and specificity. Results of the CNNs were compared to human observers' stress ratings on the same datasets (= images) of chickens using the Stressed Chicken Scale.

We found that AI can detect discomfort in individual chickens in side-view images, comparable to humans. Our findings show that certain CNN models, in particular variants of EfficientNet, show high performance in identifying stress signs in chickens. These results highlight the potential of deep learning for automated animal welfare monitoring.

To enhance model interpretability, we used a Grad-CAM, which provides valuable insights into the decision-making process of the models. We found that the AI “looks” at specific body parts of the chickens when making decisions.

This research contributes to the development of innovative, non-invasive methods for monitoring chicken welfare, and may provide the foundation for a useful tool for early detection of stress and discomfort indicators in chickens at individual animal level.

## Introduction

Artificial intelligence (AI), computer vision (CV), and machine learning (ML) are promising tools for assessing animal wellbeing (McLennan and Mahmoud, 2019; Feighelstein et al., 2023; Kahnau et al., 2023). Algorithms have been developed for pain assessment in cats (Feighelstein et al., 2023) and sheep (Zamansky et al., 2023), as well as for welfare assessment in laboratory animals (Andresen et al., 2020). In addition, AI is a cornerstone of Precision Livestock Farming, an emerging management tool for farm animals, including poultry. Precision Livestock Farming uses electronic tools such as cameras, microphones and sensors to monitor and manage livestock. It is based on data often generated by various systems and analyzed by AI (Depuru et al., 2024; Li et al., 2020; Neethirajan, 2022; Okinda et al., 2020). AI has the potential to reduce operational costs and enhance animal health through the automation and optimization of processes, including disease detection and reducing pathogen spread, although more research is needed here (Taleb et al., 2024).

The deployment of large-scale equipment, e.g., sensors and cameras, is financially prohibitive, and the integration of artificial intelligence necessitates the training of personnel and, when necessary, the modification of operational protocols. Consequently, widespread utilization remains impractical by now, and only a fraction of the potential of AI in agriculture is currently being exploited, despite the availability of certain systems in the market (Hesse et al., 2024). For poultry these include, for example, systems to identify sick animals (Okinda et al., 2019), detect lameness (Aydin et al., 2015; Li et al., 2020; Silvera et al., 2017) or bumblefoot disease (Bist et al., 2024). Other approaches aim to measure group body weight (Amraei et al., 2018, 2017), monitor behavior (Fang et al., 2021), and track chickens within the flock (Neethirajan, 2022; Ruhnke et al., 2019). Some utilize image analysis to identify potential diseases through fecal samples (Degu and Simegn, 2023), or thermographic imaging (Sadeghi et al., 2023).

While some systems are limited to experimental setups, such as birds passing through test corridors for lameness (Aydin et al., 2015) or disease (Okinda et al., 2019), others focus on detecting immobile birds within a flock (Zhuang and Zhang, 2019). However, these systems often detect health issues at a later stage, once symptoms have already manifested. A more animal-friendly approach would be to enable the early detection of chickens’ discomfort on an individual basis. Unfortunately, the observation of individual animals is still in its infancy (Hesse et al., 2024). The current study aims to contribute by developing an AI-based system focused on early detection of stress and discomfort in poultry on an individual level.

In a previous study, we developed a Stressed Chicken Scale (SCS) as a tool for human observers to assess stress in adult laying hens and dual-purpose breeds on a single animal level (Schlegel et al., 2024). The scale includes 7 body signals that indicate discomfort, distress, pain, or illness. The SCS proved to be effective to easily teach human observers to identify compromised chickens. But as chickens, like other prey animals, hide any signs of weakness (like pain or a disease) for as long as possible (Doneley, 2016), the development of an automated, standardized tool may prove to be more sensitive to detect even minor changes in animal appearance. Thus, an AI-driven system may assist in the identification of stress and discomfort in chickens in stages and situations where humans are not yet capable of doing so. A particular challenge in developing such a system is the relatively small amount of data available for such an endeavor due to the subject matter of the research. Unlike in many other applications of ML / AI, where it is relatively easy to provide the very large amounts of data needed to train most ML / AI models, such large amounts of data are typically not available in the veterinary field (see Zamansky et al. (2023)). Consequently, we opted for transfer learning (Gupta et al., 2022), and data augmentation (Shorten and Khoshgoftaar, 2019) to achieve good results even with the relatively small amount of data available.

**Fig. 1:**
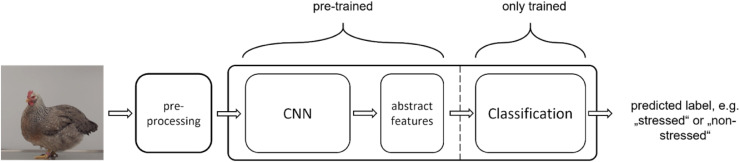
**Transfer learning for computer vision.** The image was preprocessed, in our case the image was resized, and each pixel normalized. For training, a data augmentation was added to the pre-processing step. Then a pre-trained CNN, such as *mobilenet*, or *efficientnet,* was used to create an abstract feature space. After that, a classification model was used, in our case either a logistic model, or a shallow neural net where the final layer was again a logistic model. Only the classification model was trained on our data.

**Fig. 2:**
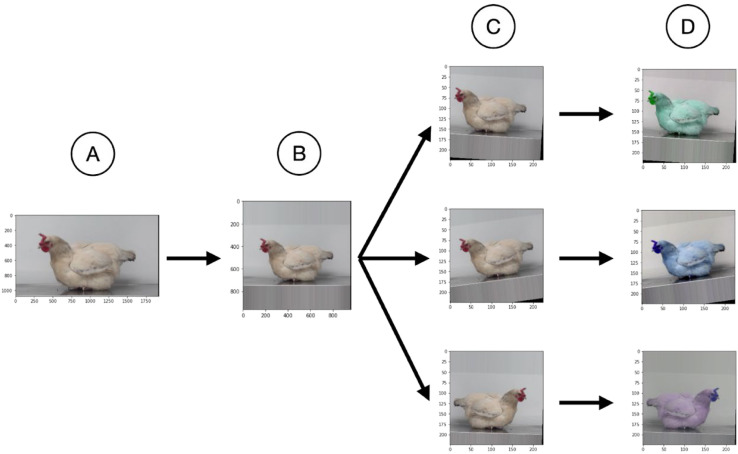
**Preprocessing and data augmentation for training.** (A) First, the images were extracted from the video frames. (B) All CNNs expect a 1:1 aspect ratio, so the images were adjusted in their aspect ratio by padding with mode ‘edge’. Additonally, the resolution was reduced. (C) Then, for the data augmentation first geometric transformations were done: each image was randomly mirrored, randomly rotated by plus or minus 10 degrees, a random image was cut out and the resolution was reduced to 224 × 224 pixels; possible results are shown. (D) Then for the photometric transformations, brightness, contrast, hue, and saturation were randomly changed; possible results are shown. As the final step (not shown in this figure), the data type was changed to 32bit float with each color channel of each pixel scaled to the range from zero to one, and then lastly normalizing each color channel.

In computer vision, image classification models usually consist of two parts: the first part is the convolutional neural network (CNN), which generates from the unstructured input data (i.e., the provided image) a set of structured, but abstract, features. The second part then performs the actual classification using these abstract features generated by the CNN from the input data (Fig. 1). Instead of training the CNN, pre-trained computer vision CNNs are used, and only the classification part of the model needs to be retrained. This approach is known as *transfer learning*, which is a suitable method for training an image recognition model given the small size of the data set (Gupta et al., 2022; Weiss et al., 2016). Transfer learning uses pre-trained models, which avoids the need to completely retrain an entire model with its often millions of weights. Instead, only a very small part of the model is retrained, which drastically reduces the amount of training data required. Data augmentation can be used to increase the amount and variance of available image data. This can be achieved through relatively simple geometric and photometric image transformations, for example (see Fig. 2).

The abstract structured feature set created by the CNN is not easy to interpret, but techniques such as the Grad-CAM (Selvaraju et al., 2017) can be used to determine which parts of the image have been used by the model for the respective class predictions.

The aim of this study was to investigate whether AI can detect signs of chicken discomfort and, if so, whether it can do so more precisely than human observation. Additionally, we sought to determine if training an AI tool with small data produces satisfactory results. The “Happy Chicken Tool” was developed for this purpose.

## Material and methods

### Animals for training data (D)

The video footage used as training dataset was collected for the study by Schlegel et al. (2024). The footage features chickens of various ages and breeds, including young and adult hens, as well as young roosters up to five months old, kept by private owners. The chickens were from laying lines, including brown and white layers, as well as dual-purpose breeds such as Amrock, Bielefelder Chicken, Bovans Black, German Empire Chicken, Rhode Island Red, German Cuckoo, Sussex, and Vorwerk Chicken. The videos were taken during routine vaccinations at the Institute of Poultry Diseases, Freie Universität Berlin, Germany between December 1, 2020, and September 7, 2021, throughout the day (10 a.m. to 5 p.m.). Vaccinations, and therefore filming, took place on demand, with some weeks in between.

The vaccination was chosen as a stressor to induce signs of discomfort in the animals, as shown in the study design by Schlegel et al. (2024).

**Fig. 3:**
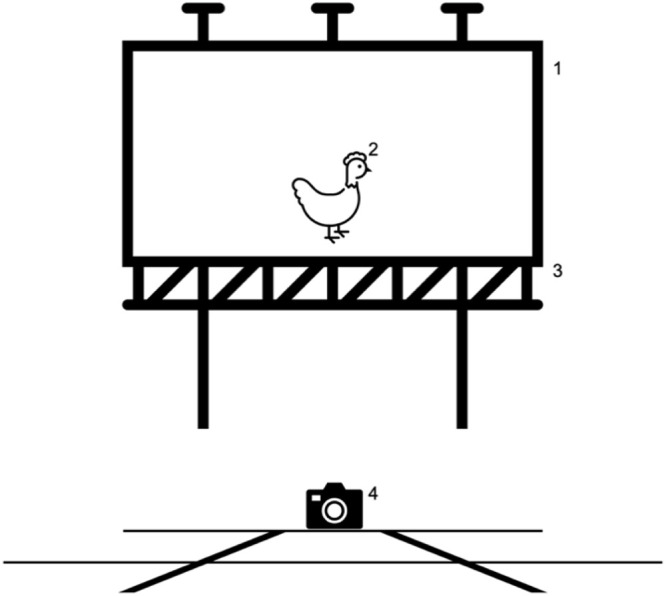
**Experimental setup.** A white screen (1) was placed directly behind the chicken (2) standing on the extermination table (3). A video camera (4) was set up with a lateral view of the chicken. (Icon created using the Pictogram feature of MICROSOFT Word (Microsoft Office 365).

Videos were processed using iMovie for iOS and macOS (APPLE Inc., Cupertino, CA). The dataset included 113 videos, with 64 videos of stressed chickens and 49 videos of unstressed chickens. Videos length was between 1 and 18 seconds, with a mean of 2 seconds. There was a total of 3,982 frames for the "stressed" class and 4,195 frames for the "unstressed" class. The videos showed the chickens in their entirety from a lateral perspective (Fig. 3) with or without clear body signals of stress according to the findings of Schlegel et al. (2024). Side view was mandatory because some of the body signals described in the SCS could only be clearly assessed from a lateral view. Only videos in which the chickens showed clear signs of being either stressed or unstressed were used for training. Chickens were classified as either stressed or unstressed according to the Stressed Chicken Scale by Schlegel et al. (2024) by a human expert (LSP). "Stress" was defined as "any physical discomfort such as pain or illness, as well as emotional distress caused by 'all feelings of displeasure not covered by the exact concept of pain'” (Bernatzky, 1997).

### Animals for 1st validation (V1)

140 chickens (Isa brown, female, 36 weeks) were filmed (SONY HDR-PJ 650 Handycam) at the Aviforum Zollikofen, Switzerland, on January 22, 2024 during data collection involving catching out of an aviary barn and crating prior to handling in an animal experiment (animal experimentation permit given by the Cantonal Veterinary Authority Bern (“Amt für Veterinärwesen”): BE33/2023). After overhead weighing, blood sampling, and overhead radiography, the hens were placed on the floor. One chicken at a time was filmed sitting on the ground in front of a gray door in a barn vestibule, with the camera positioned sideways at the chickens' eye level. To achieve greater data variance, this setting was chosen to create more realistic imaging conditions, e.g. more realistic lighting conditions.

Screenshots (V1_chicken_ = 140) were taken from this video footage immediately after both feet reached the ground (hen standing alone), when the hen was viewed in its entirety from the side. Chickens were categorized as either stressed or unstressed by LSP according to the findings by Schlegel et al. (2024) and included *n* = 52 stressed and *n* = 88 unstressed chickens. This dataset was used for early stopping (Prechelt, 2012), a method to prevent overfitting by ending the training of the classification layer with the training dataset D.

Early stopping was implemented so that, after *n* training rounds, the trained model would be validated with the V1 dataset. If the new model had a better value for the statistic of interest (Cohen’s kappa) than the previous best model, it would be saved. Training continued thereafter. If no better model was found, the last best model was set as the final model of the training.

### Animals for 2nd validation (V2)

59 adult hens from laying lines (including brown and white layers) and dual-purpose breeds (Bielefelder Chicken, Bovans Black, German Cuckoo, German Empire Chicken, Marans, Rhode Island Red, Sussex, and hybrids) were filmed under the same conditions as the chickens for the Training data (Schlegel et al., 2024), as shown in Fig. 1, from October to December 2023. Filming took place again on demand throughout the day (10 a.m. to 5 p.m.), with some weeks in between. This procedure can be considered a routine non-experimental clinical veterinary practice, according to the Berlin State Authority (“Landesamt für Gesundheit und Soziales”), position statement number (“StN”) 036/23).

A screenshot of each video (1 video = 1 chicken) was taken immediately after the chicken was placed back on the table after being vaccinated, when the chicken was seen from the side in its entirety. Screenshots (V2_chicken_ = 59) were categorized as either stressed or unstressed by LSP according to the findings by Schlegel et al. (2024) and included *n* = 33 stressed and *n* = 26 unstressed chickens.

### Evaluation by people

Screenshots from the training dataset (D_chicken_ = 80), first validation dataset (V1_chicken_ = 140) and second validation dataset (V2_chicken_ = 59) were scored with the Stressed Chicken Scale (SCS) by human observers (D_human_ = 20, V1_human_ = 15, V2_human_ = 11) and classified as either stressed or unstressed. The assessment was conducted individually, with no opportunity to communicate with other observers in the group.

The SCS includes 7 body signals in a lateral view: a dropped tail, a tucked-in head, (partially) closed eyes, an (partially) open beak while breathing, bent legs / sitting or lying down, dropped wings, and ruffled plumage.

Observers were different students of veterinary medicine (D_human_ = 20, V1_human_ = 13, V2 _human_ = 11) in their final year of education at the Freie Universität Berlin and animal care trainees (V1 _human_ = 2). They received a 20-minute joint training session with sample pictures on how to use the SCS via a Microsoft PowerPoint (Microsoft Office 365) presentation recorded by LSP.

### Software and hardware used

For training and validation PyTorch (torch 2.6.0, torchvision 0.21.0, CUDA 12.6), PyAV (version 14.1.0), Python (version 3.11.7), and Spyder (version 6.0.1, standalone, IPython version 8.31.0) were used. Training was performed on an Intel i9-14900F CPU, 64 GB RAM, NVIDIA GeForce RTX 4090 graphic card with 24 GB GPU memory, and NVMe 3500 Micron 2048 GB drive.

PyTorch offers a variety of different pre-trained CNNs for image classification. After initial screening, the CNN families used were MobilNet v3 (3 members) (Howard et al., 2019), EfficientNet (8 members) (Tan and Le, 2019), EfficientNet v2 (3 members) (Tan and Le, 2021), and Resnet (5 members) (He et al., 2016). These pre-trained models use weights obtained by the CNN provider through training on the ImageNet dataset (Stanford-Vision-Lab, 2020). Whenever possible, the latest version of the trained weights was used.

**Fig. 4:**
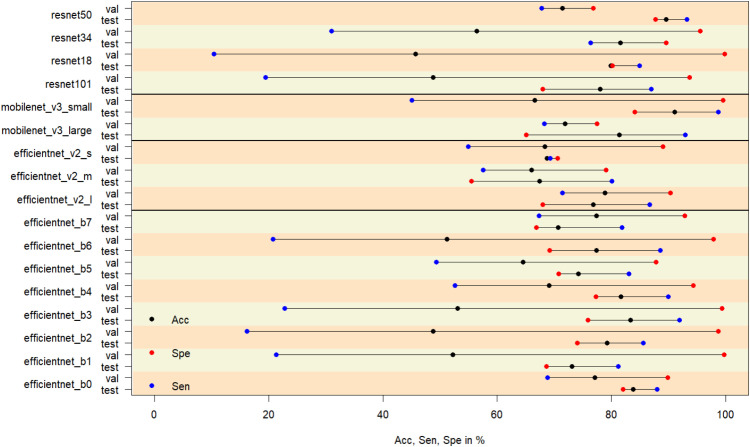
**Initial CNN screening.** Different pre-trained CNN architectures were tested and the best selected. Different CNN families were trained with the training dataset and the accuracy, sensitivity, and specificity were measured in a 10-fold repeated cross-validation, and with a different validation dataset. CNNs with the highest values of all three measures and for both data sets were selected. The initial best CNNs were *efficientnet_b0*, and *efficientnet_b7, efficientnet_v2_l*, and *mobilenet_v3_large*. These CNNs were then further investigated.

Other tested CNN families or individual models included *AlexNet, GoogLeNet, MNASNet, RegNet*, and *ShuffleNet*. However, in initial tests, these did not demonstrate the same level of performance as the selected models (see Fig. 4), and further investment was not pursued. The *ConvNeXt*, and *DenseNet* families were not included.

For the Grad-CAM, the package grad-cam (version 1.5.4) was used (Gildenblat and contributors, 2021).

### Preparation of the training data

The training data (D) were color videos either in HD (1280 by 720 pixels, 16:9 aspect ratio, 8bit), or in full HD format (1920 by 1080 pixels, 16:9 aspect ratio, 8bit) with 24 frames per second. The videos were read frame-wise (in total: stressed: 3,982 frames; unstressed: 4,195 frames).

Since all CNNs used expected an image with an aspect ratio of 1:1 as input, so-called padding was used with mode 'edge'. In this mode, the pixels at the edge of the longer side of the image are extended into the padding area (see Fig. 2), resulting in an image of 1280 × 1280 pixels, or 1920 × 1920 pixels. To reduce the memory pressure, the resolution is reduced to 960 × 960 pixels. Each image then uses about 22 megabits (about 2.8 MB) of memory (three color channels * 960 * 960 * 8 bits). No further resolution reduction was performed, as this would have resulted in artifacts in the subsequent data augmentation steps, which are critical for training.

For training, image augmentation was used after the initial preprocessing to increase the variance of the image information. The following protocol was applied for the augmentation procedure (see also Fig. 2):

1. On average, every second image is horizontally mirrored,
2. the image is randomly rotated in the ± 10° range,
3. the image is reduced to a resolution of 256 by 256 pixels, and a 224 by 224 pixels image is randomly cropped from this image, causing a random shift of the image relative to the original image,
4. finally, the image is randomly modified in terms of brightness (range: 0.875, 1.125), contrast (range: 0.5, 1.5), saturation (range: 0.5, 1.5) and hue (range: −0.05, 0.05). Here, the standard values provided by PyTorch have been used; other values did not result in any improvement.

For all CNNs, images with a resolution of 224 by 224 pixels were used as input. Additionally, the values of each color channel of each pixel must be scaled to a range from zero to one (as a 32-bit floating point number), and then the scaled value of each color channel must be normalized using values obtained for the ImageNet images (mean = [0.485, 0.456, 0.406], standard deviation = [0.229, 0.224, 0.225]). Normalization was done as:

$$z=\frac{x-mean}{sd}$$

### Preparation of the validation data (V1 and V2)

The validation images were color images with different pixel count (range from 1200 by 1000 pixel to 1700 by 1600 pixel, 8bit), and then padded to a 1:1 aspect ratio with mode ‘edge’ as the training images. They were then resized to 224 by 224 pixels. Then, each color channel of each pixel was scaled to a range from zero to one (as a 32-bit floating point number), and then each color channel was normalized as the training images.

### Preparation of computer vision models

The CNN of each model was ‘fixed’ (also called ‘frozen’) for training, ensuring that the CNN weights remained constant during the training process. PyTorch provides pre-trained weights for each CNN, determined by training on the ImageNet dataset (IMAGENET1K_V1, or IMAGENET1K_V2, if available). Most models used a simple multi-nominal model as the last layer for classification, only a few (namely *mobileNet_v3*) used a neural network for classification. The original classification model of each CNN was designed to classify 1000 classes, while here we have only two. The classification layer was removed and replaced by either a simple logistic model or a shallow neural net (only one hidden layer, ReLU activation function), while the last layer again was a simple logistic model.

### Implementation of training

For each iteration step of the gradient descent, a new small sample, the mini-batch, was randomly drawn from the training data, as described in the following. For training, mini-batches of 100 to 300 preprocessed and augmented images were used, with the two classes contributing 50/50, for a balanced sample. Usually, the mini-batch would be sampled uniformly from each class of the entire training image dataset. However, our image dataset was created from frames of videos of different lengths, with each video representing a specific chicken. To avoid over-representing chickens from longer videos, for each class (stressed or unstressed) we first sampled one chicken (i.e., one video) uniformly, and then sampled one frame uniformly from the frames of the corresponding video. For this purpose, a new train loader function had been programmed.

Given that the classification model is a logistic model, the Binary Cross Entropy Loss (BCEloss) was used as the loss function.

For the gradient descent method, either the method *Adam* (adaptive moment estimation), or *RMSprop* (Root Mean Squared Propagation) was used (Kingma and Ba, 2017; Tian et al., 2023). To create the final models, the models were trained using the entire training dataset (D), while the V1 dataset was used as a validation dataset for early stopping (Prechelt, 2012).

For cross-validation, as used for the screening of CNNs, one or more whole videos (each representing a single chicken) were used as a hold-out dataset. The minibatch was drawn from the remaining videos as described above. The entire hold-out dataset was used for testing.

### Grad-CAM

For the Grad-CAM, the original model was loaded, the classifier removed and replaced with the new classifier selected. The weights of the trained model were then loaded into the model. The model was then ‘fixed’, so that no weight changes occurred during the Grad-CAM-process. Image preparation was the same as for the validation images.

For the Grad-CAM, the very last layer of the CNN was used, just before the abstract features were fed into the classification model. The Grad-CAM produces its result for the output chosen by the user, here either the stressed, or unstressed class. For better comparison, we created the Grad-CAM output for both classes, together with the model output, the percentage certainty that the presented image represents a stressed chicken.

The package used provides a wide variety of different types of cams; we used the Grad-Cam, and the HiRes Grad-CAM.

### Statistics

Statistical analyses were performed using SPSS Statistics, version: 26 (IBM, IBM Deutschland GmbH, Germany). The crosstab function was used to determine sensitivity (true positive = TP) and specificity (true negative = TN) as well as agreement scores using Cohen's kappa (κ). Human observers’ assessments on average were compared to an expert standard (= LSP’s score, taken as the truth of reference) and used to determine the interobserver agreement between the multiple observers. MICROSOFT Excel (Microsoft Office 365) worksheets were used to record the scores. In accordance with Landis and Koch (1977) the following cut-off values were used to assess kappa values: <0.00 = less than chance (poor) agreement, 0.00-0.20 = slight, 0.21–0.40 = fair, 0.41–0.60 = moderate, 0.61–0.80 = substantial, 0.81–1.00 = almost perfect.

Prediction quality of the human observers and computer vision models were measured by accuracy (Acc), sensitivity (Sen), specificity (Spe), and Cohen's kappa (κ). P-values <0.05 were considered significant. Additionally, we computed the 95 % confidence interval for accuracy, sensitivity, specificity and Cohen’s kappa for the V1 and V2 datasets, since the sample size of both datasets is rather small with *n* = 140, and *n* = 59, respectively. If the confidence interval for the accuracy does not include the no-information criterion, then the prediction is better than simply predicting the most prominent class. The no-information criterion is the frequency of the number of images of the most prominent class divided by the total number of images. The confidence intervals for accuracy, sensitivity, and specificity were computed using a beta distribution, using the *beta.interval* function from the SciPy package (version 1.15.1). The accuracy was computed with α=TP+TN+1 and β=FP+FN+1; the sensitivity was computed with α=TP+1 and β=FN+1, and for the sensitivity with α=TN+1 and β=FP+1.The confidence interval for kappa was calculated using the standard error for kappa (as specified in equation 13 by Fleiss et al. (1969)) and the usual assumption of a normally distributed confidence interval:

κ95%CI=κ±1.96×SE(κ)

## Results

### Screening the selected model families

First, we screened the selected CNN families with a 10-fold cross-validation (Kohavi, 1995) with the Adam optimizer, a step size of 1e-2, no weight decay, 200 iterations per cross-validation, and a logistic model as classifier for each model. The weights of the classifier were initially set randomly, but the weights were carried over to each new cross-validation round, so we had a warm-start for each model after the first cross-validation round.

For each round of cross-validation, accuracy, sensitivity, and specificity were computed for the omitted data of the training data (also called test data), and for the V1 data set. These statistics were averaged and plotted (see Fig. 4). We selected the models, for which the three statistics appeared to be the best for the test and validation data. These models were *efficientnet_b0, efficientnet_b7, efficientnet_v_l*, and *mobilenet_v3_large*. Except for the first model, all other models are the largest member of their family.

### Refining the winning models

The selected models were then refined*.* This was done by trying to replace the simple logistic model with a shallow neural net (one hidden layer with ReLU activation function), while the output layer was again a logistic model. We tried various numbers of nodes in the hidden layer (from 2 to 1000), and with different values for the weight decay, and different optimizer routines (Adam and RMSprop). We trained the model for 50 or 100 iterations with a balanced sample (per chicken) of 200 augmented images from the training dataset per iteration. We then computed the test statistics for the training dataset, and the V1 dataset. A model was considered good, if the kappa for both datasets became the largest achievable. The best model was found for the *efficientnet_v2_l* architecture with a shallow neural net (hidden nodes = 1000) for the classifier, a weight decay value of 1e-5, and using the optimizer Adam. Final results see Table 1.

**Table 1:** **Quality statistics.** Quality statistics for comparing humans and machines. For data sets V1 and V2 the 95 % CI intervals for the accuracy (Acc) are given, since the sample size is rather small. If the CI does not include the no-information criterion (nic), then the Acc is statistically significant and the prediction is better than just predicting the most prominent class, as a naive predictor would do.

		**Dataset**											
		**Training data (D)**				**Validation 1 (V1)**				**Validation 2 (V2)**			
		**Cohen’s Kappa**	**Acc**	**Sen**	**Spe**	**Cohen’s Kappa** _(__95__% CI)_	**Acc** _(nic;__95__% CI)_	**Sen** _(__95__% CI)_	**Spe** _(__95__% CI)_	**Cohen’s Kappa** _(__95__% CI)_	**Acc** _(nic;__95__% CI)_	**Sen** _(__95__% CI)_	**Spe** _(__95__% CI)_
**Method**	**Human observers**	0.66	0.85	0.86	0.84	0.57	0.80	0.97	0.63	0.75	0.88	0.89	0.87
	**efficientnet b0**	0.804	0.91	0.92	0.91	0.568 _(0.387-0.750)_	0.79 _(0.63; 0.71-0.84)_	0.86 _(0.75-0.93)_	0.74 _(0.64-0.82)_	0.507 _(0.236-0.778)_	0.76 _(0.56; 0.64-0.85)_	0.88 _(0.72-0.95)_	0.61 _(0.42-0.78)_
	**efficientnet b7**	0.755	0.89	0.88	0.95	0.676 _(0.489-0.863)_	0.84 _(0.63; 0.77-0.90)_	0.88 _(0.77-0.94)_	0.82 _(0.72-0.88)_	0.670 _(0.377-0.962)_	0.83 _(0.56; 0.71-0.91)_	0.70 _(0.52-0.83)_	1.00 _(0.87-1.00)_
	**efficientnet v2 l**	0.863	0.94	0.93	0.94	0.715 _(0.527-0.903)_	0.86 _(0.63; 0.79-0.91)_	0.86 _(0.75-0.93)_	0.86 _(0.78-0.92)_	0.702 _(0.406-0.997)_	0.85 _(0.56; 0.73-0.92)_	0.73 _(0.56-0.85)_	1.00 _(0.87-1.00)_
	**mobilenet v3 large**	0.662	0.83	0.76	0.94	0.565 _(0.384-0.746)_	0.79 _(0.63; 0.71-0.84)_	0.85 _(0.72-0.92)_	0.75 _(0.65-0.83)_	0.519 _(0.245-0.793)_	0.76 _(0.56; 0.64-0.85)_	0.79 _(0.62-0.89)_	0.73 _(0.54-0.86)_

According to the PyTorch documentation for the model *efficientnet_v2_l*, the input size for the image should be 480 by 480 pixels with normalization values of 0.5 for the mean and the standard deviation for each color channel, instead of the value we used. We also needed to increase the resize value in step 3 of the image augmentation process to 548 pixels. Due to the larger size of the images, we had to decrease the batch size to 100 images. Using these documented values for image input size and the normalization did not improve the predictability over the best model we found previously, but it did slow down the training and validation process due to the larger image sizes. We therefore discarded this and used the smaller size of the input image and the normalization as described in the Methods section.

### Human observers compared to AI

Training data (D): There was substantial agreement between the human observer's overall rating and the expert rating (κ = 0.66, Acc = 0.85, Sen = 0.86, Spe = 0.84), when using the SCS. Interobserver reliability was found to be moderate (κ = 0.53). The trained model with the CNN *efficientnet_b0* (10fold CV; κ = 0.804, Acc = 0.91, Sen = 0.92, Spe = 0.91) and *efficientnet_v2_l* (10fold CV; κ = 0.863, Acc = 0.94, Sen = 0.93, Spe = 0.94) gave the best models of the selected CNNs. Both even significantly outperformed human observers (*r* = <0.001 and *r* = 0.021, respectively).

Validation 1 (V1): There was moderate agreement between the overall rating of the chickens as stressed or unstressed by the human observers using the SCS and the rating provided by the expert (κ = 0.57, Acc = 0.80, Sen = 0.97, Spe = 0.63). Additionally, the interobserver reliability was moderate (κ = 0.58). From the selected CNNs *efficientnet_b7* (κ = 0.676, Acc = 0.84, Sen = 0.88, Spe = 0.82) and *efficientnet_v2_l* (κ = 0.715, Acc = 0.86, Sen = 0.86, Spe = 0.86) performed best, and even better than the human observers, although not statistically significant better (*r* = 0.175 and *r* = 0.362).

Validation 2 (V2): There was substantial agreement between the overall rating of the chickens as stressed or unstressed by the human observers using the SCS and the rating provided by the expert (κ = 0.75, Acc = 0.88, Sen = 0.89, Spe = 0.87). Interobserver reliability, too, was found to be substantial (κ = 0.62). Again, *efficientnet_b7* (κ = 0.670, Acc = 0.83, Sen = 0.70, Spe = 1.00) and *efficientnet_v2_l* (κ = 0.702, Acc = 0.85, Sen = 0.73, Spe = 1.00) performed best among the selected CNN models. But again, they did not perform statistically significantly better than humans (*r* = 0.530 and *r* = 0.524, respectively).

Due to the sample sizes of V1 (*n* = 140) and V2 (*n* = 59), the 95 % confidence intervals for the statistics used are included in Table 1. If the confidence interval for the accuracy does not include the no-information criterion, then the model's prediction is statistically significantly better than just predicting the most prominent class.

We conclude that discomfort of individual chickens in side view images can be detected by AI with a reliability comparable to humans, with *efficientnet_v2_l* giving the best results for all three data sets. However, it should be noted that the prediction quality statistics for the three datasets were obtained in different ways for the computer vision models. For dataset D, 10-fold cross-validation with a fixed number of training iterations was used. This was relevant for screening suitable CNNs. For the development of the final model on the selected CNNs, the entire dataset D was then used for training and dataset V1 was used as a validation data set for early stopping. Dataset V2 was then used for independent validation of the model.

### Grad-CAM

We found that the chicken itself plays a critical role in the AI's decision making, more so than the chicken's surroundings. We see a tendency for the AI to “look” at the chicken's caudal body, especially the tail, the legs and the wings, when classifying the chicken as stressed. When deciding on unstressed, the AI “looks” at similar body parts, but more often at the wings and cranial body parts (see Table 2).

**Table 2:** Grad-CAM. HiRes Grad-CAM heatmap images for the same sample images from the three datasets as shown in Fig. 5 for both classes (stressed / unstressed), giving an idea of where the AI is "looking" when making its decision, including the prediction rate (P) for the class selected.

## Discussion

We were able to build several computer vision models that could either outperform human observers for distinct set-ups (dataset and type of human observers) or at least compete with them (see Table 1). But interestingly, none of the computer models, and none of the human observers were able to perform a perfect prediction, indicating overlap in the features representing the stressed and unstressed chickens. This could indicate that the binary classification into stressed and unstressed may be too crude. Perhaps, a finer subdivision into three classes (stressed, intermediate, unstressed) could help. A similar approach is widely used to assess pain in laboratory animals such as mice, where a grading from “Not present” to “Moderate” to “Severe” is used (Langford et al., 2010). Something similar should be considered and further studied in chickens.

Although we tried many different computer vision models, only a few, and in the end only one model, *efficientnet_v2_l*, performed by far the best on all three datasets in terms of Cohen’s kappa. This model is already a large CNN (the model has more than 118 million parameters and takes about 450 MB of disk space), but so is the second best model, *efficientnet_b7*. In general, the predictive quality of the models for our chickens seems to correlate with the accuracy specified in PyTorch with regard to the ImageNet-1 K dataset (where acc@1 is the classic accuracy relevant to us). The models' reported accuracies are 75 % for *mobilenet_v3_large* (Torch-Contributors, 2017-2025d), 78 % for *efficientnet_b0* (Torch-Contributors, 2017-2025a), 84 % for *efficientnet_b7* (Torch-Contributors, 2017-2025b), and 86 % for *efficientnet_v2_l* (Torch-Contributors, 2017-2025c).

We believe that a more specific CNN for our purposes could be smaller and better performing in terms of prediction and speed. Smaller prediction models would also be advantageous, because they would run much faster on less powerful consumer hardware (e.g., smartphones or tablets). This would be more user-friendly and may lead to better acceptance for widespread use.

Due to the amount of data to be expected for such an endeavor, either more image material would have to be created, or data would have to be artificially generated using more sophisticated augmentation techniques than those used here.

**Fig. 5:**
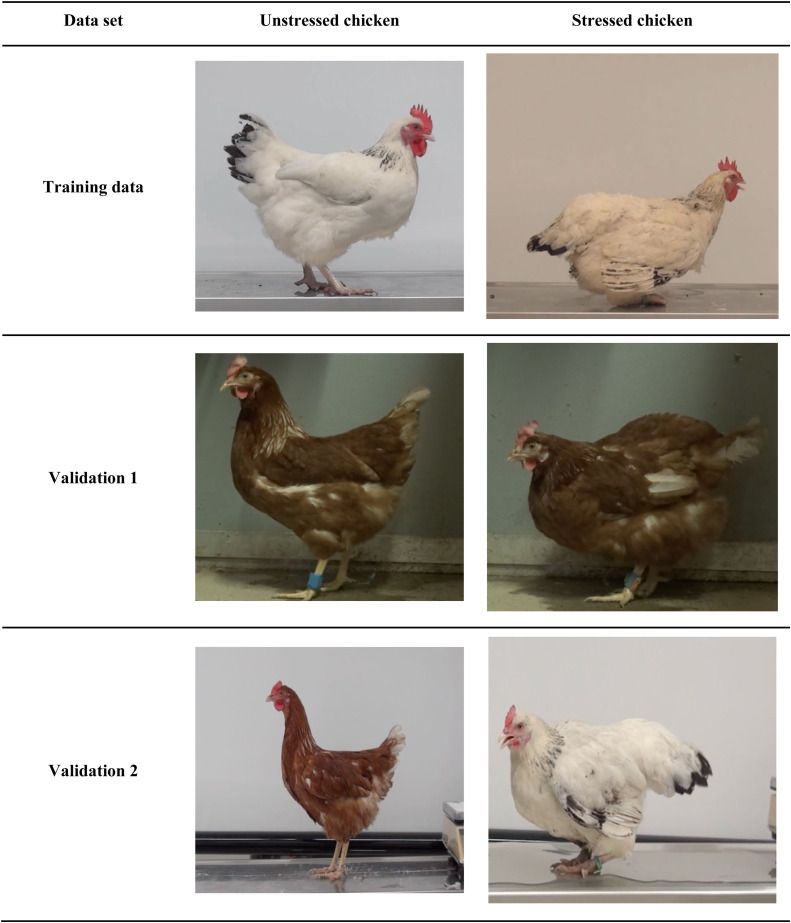
**Example images for both classes (stressed / unstressed) for each data set used.** Example images from the Training data, Validation 1, and Validation 2 to provide insight into the variability of chicken breeds, feather color, and different environments, such as lighting conditions, in the data sets.

The variance in the different datasets was a complication for the computer models. The training dataset is rather uniform and clean in the background (see Fig. 3), with a wide variety of chicken breeds. Dataset V1 has a rather dark background with corresponding darker chickens, which is not represented in the Training dataset (see Fig. 5). Through data augmentation, especially photometric transformations, we were able to increase the predictive power for the V1 dataset. We also used dataset V1 for early stopping of the training of the model, which was very important for the success of the training. However, compared to the other datasets, both humans and AI scored slightly lower with Cohen’s Kappa on the V1 dataset. We suspect that this is due to the lighting conditions, which are critical not only to computer models but also human decisions. Di Giminiani et al. (2016) found something similar in humans in their study of the Piglet Grimace Scale, in which a lamp cast a shadow that affected humans' judgments of the piglets' level of pain in the pictures. Furthermore, people are not necessarily consistent in their decisions, which can depend on a variety of conditions (age, gender, experience, mood effects, etc.), some of them can vary from day to day, known as cognitive bias / human error, as described for some animal assessment systems (Orth et al., 2020; Schlegel et al., 2024). A computer model should not have these limitations (Guo et al., 2023) and should allow for consistent evaluation results if stable image quality is provided. Our study design did not include the collection of personal data (such as age, gender, educational background, etc.) from the human participants.

In dataset V2, parts of other objects (e.g., parts of the scale used to weigh the chickens) were visible, that were not visible in the training dataset. This could have misled some of the computer models. We might increase the predictive power either by merging all available data, or by more advanced data augmentation techniques for the training dataset, such as random erasing, or mixing images. However, we conclude that an object detection with classification would be the logical next step. This would allow the model to find and classify the object of interest in the image, independent of other visible objects. It may also be possible to directly capture the features of different parts of the chicken's body and use them for classification. The computer vision models we have investigated so far use the whole image for classification, but this is the first step needed for any further development.

Since chickens are flock animals, object detection can also simplify image acquisition, by eliminating the need to separate the animals first. However, individual animal identification in large flocks is still a key issue in poultry (Hesse et al., 2024) and is part of current research using e.g. deep learning models based on You Only Look Once (YOLO) (Guo et al., 2023; Neethirajan, 2022).

To help us understand which parts of the image were used by the model to make the prediction, we used a Grad-CAM, similar to research approaches on automated pain recognition in cats (Feighelstein et al., 2023). We believe that the Grad-CAM image and the predicted percentage that the model classifies the image as stressed or unstressed should be used together as the output of the model, as it provides a form of justification for the model's decision. In this way, it could serve as a form of quality control for the model, which should be interpreted responsibly by humans in context. Nevertheless, the Grad-CAM indicates a difference in how the computer model makes the decision, compared to the developed scale. The computer model uses much less of the available image of the chicken, and therefore rejects some features of the Stressed Chicken Scale. This leads to the question of whether anything would change if we specified the region of interest for the machine. In our setting, the machine was ‘free to look wherever it wanted’ (i.e., use the whole image), while the humans had seven specified body parts on the animals to look at, given by the SCS. More research needs to be done to investigate this further.

Even when using transfer learning, existing frame works and Application Programming Interfaces (APIs), developing good models in deep learning is still a rather laborious task. This applies not only to the training process to obtain good performing models, where a lot of obvious and hidden so-called hyperparameters need to be fine-tuned (includes data augmentation), but also to the collection of important information about the pre-trained models or methods. Unlike in other fields, important research results for deep learning are often not published in peer-reviewed journals, but remain published as pre-prints, in particular on arXiv.

The interdisciplinary collaboration between domain experts, in this case veterinarians, and technological experts in ML / AI has made the successful development of the CV models presented here and their evaluation possible. We have put a lot of emphasis on validation with differently generated datasets. However, we are aware that the model is in fact a diagnostic test and that, for a practical application of the model, a validation via a ring trial would be the better method of validation. But, the effort of a ring trial should only be attempted if the computer vision model can be improved beyond its current state, which is by now quite comparable to other AI models applied to animals (see Table 1). Feighelstein et al. (2023), for example, found an accuracy for a deep learning approach with *ResNet50* in cats of about 65 % on a diverse dataset (multi-breed, multi-sex), and over 72 % on more homogenic data (female, mixed domestic short hair) (Feighelstein et al., 2022). One example of deep learning systems being used with chickens is an approach for the early detection of bumblefoot in hens that uses YOLO, among other methods (Bist et al., 2024).

The next step in using computer vision to improve chicken welfare would be the object recognition with classification, as mentioned above. Furthermore, the chickens were not only depicted individually, but were also always shown from the side and in front of a neutral background for the design of the stress scale. For a more practical application, other perspectives and backgrounds should be considered. At the same time, a detailed analysis of the Grad-CAM results, which is still pending, may help to develop an improved and more robust scale for detecting discomfort in chickens. The current analysis highlights the need for greater consideration of intermediate stress states in these animals. It is also clear that the stress state of a chicken is a continuous variable, and the binary classification into two classes appears to be an oversimplification.

## Conclusions

In this interdisciplinary collaboration we were able to successfully create computer vision models for classifying chicken images into stressed / unstressed chickens (“Happy Chicken Tool”). These models were able to detect discomfort in chickens with a level of accuracy comparable to that of human observers using an expert-developed scale. The computer models and the human observers used different features of the chickens to make their respective decisions. Our results will be used to improve both, the scale and the computer vision models. In this way, we hope to make a significant contribution to improving animal welfare.

## CRediT authorship contribution statement

**Larissa Schlegel-Pape:** Writing – review & editing, Writing – original draft, Visualization, Validation, Resources, Methodology, Investigation, Formal analysis, Data curation, Conceptualization. **Robert Opitz:** Writing – review & editing, Writing – original draft, Visualization, Validation, Software, Resources, Methodology, Investigation, Formal analysis, Data curation, Conceptualization. **Marko Henning:** Resources, Software, Writing – review & editing. **Cristina Ortiz Cruz:** Resources, Software, Writing – review & editing. **Anne S. Kleine:** Data curation, Writing – review & editing. **Sabine G. Gebhardt-Henrich:** Resources, Writing – review & editing. **Hans Mielke:** Conceptualization, Writing – review & editing. **Carola Fischer-Tenhagen:** Writing – review & editing, Supervision, Project administration, Conceptualization.

## Disclosures

Informed consent was obtained from all human subjects involved in the study. The authors declare no conflicts of interest and confirm that no external funding was received for the research. There are no patents, additional activities, or relationships to disclose.
